# Clinical and molecular characterization of an outbreak of leptospirosis in dogs from Los Angeles County, California, USA, 2021

**DOI:** 10.1128/jcm.00240-26

**Published:** 2026-05-26

**Authors:** Max W. Randolph, Jarlath E. Nally, Sean K. Yoshimoto, Betty Chow, David M. Wagner, Nathan E. Stone, Jason W. Sahl, Camila Hamond, Karen LeCount, Tod Stuber, Hans van der Linden, Krystle Reagan, Alexander Schrieber, Jamie Sebastian, Jane E. Sykes

**Affiliations:** 1 William R. Pritchard Veterinary Medical Teaching Hospital, School of Veterinary Medicine, University of California-Davis 70733, Davis, California, USA; 2 Infectious Bacterial Diseases Research Unit, National Animal Disease Center, USDA Agriculture Research Service 57837 , Ames, Iowa, USA; 3 VCA West Los Angeles Animal Hospital 295176 , Los Angeles, California, USA; 4 VCA Animal Specialty and Emergency Center 578040 , Los Angeles, California, USA; 5 The Pathogen and Microbiome Institute, Northern Arizona University 3356, Flagstaff, Arizona, USA; 6 U.S. Department of Agriculture, National Veterinary Services Laboratories, APHIS, Ames, Iowa, USA; 7 Department of Medical Microbiology and Infection Prevention, World Organization for Animal Health (WOAH) and National Collaborating Centre for Reference and Research on Leptospirosis, Amsterdam University Medical Center, University of Amsterdam 1234, Amsterdam, the Netherlands; 8 Department of Medicine, Epidemiology, School of Veterinary Medicine, University of California-Davis 70733, Davis, California, USA; Vanderbilt University Medical Center, Nashville, Tennessee, USA

**Keywords:** zoonosis, azotemia, *Leptospira*, microscopic agglutination test

## Abstract

In 2021, the Los Angeles County (LAC) Department of Public Health suspected a leptospirosis outbreak in LAC affecting over 200 client-owned dogs. We aimed to characterize the outbreak and describe microbiologic findings, risk factors, diagnostic test performance, and outcomes in dogs diagnosed with leptospirosis at two specialty practices. *Leptospira* culture isolates from four cases were subjected to serotyping and whole-genome sequencing (WGS); WGS was also performed on one enriched genome isolate. After the outbreak, data were gathered on 59 cases and compared to the background hospital population (controls, *n* = 15,536). All isolates were *Leptospira interrogans* serovar Canicola, but each was distinct based on WGS. Cases clustered in space and in time. Cases evaluated during the outbreak peak had increased odds of exposure to indoor congregate facilities (ICFs). None of the 47 dogs with known leptospirosis vaccination history were completely vaccinated. *Leptospira* real-time PCR on blood and urine was positive in 15/56 (27%) and 49/54 (91%) of dogs, respectively. Initial serologic testing using the microscopic agglutination test and point-of-care tests was positive in 22/29 (76%) and 27/35 (77%) of dogs, respectively. Fifty-four (92%) of 59 dogs survived to discharge; some remained azotemic. No associated human cases were identified. In conclusion, *L. interrogans* serovar Canicola was associated with a leptospirosis outbreak in unvaccinated dogs, which had public health implications given widespread dog ownership rates. Data analysis suggested multiple infection sources, including ICFs. Urine PCR was the most sensitive diagnostic test. Such outbreaks might be prevented through more widespread vaccination.

**IMPORTANCE:**

Leptospirosis is a zoonotic bacterial disease transmitted through the urine of infected animals. We characterized an outbreak of leptospirosis in unvaccinated dogs in Los Angeles County and showed that cases were associated with housing in dog daycare and boarding facilities. Culture of several *Leptospira* isolates from the post-peak period identified *Leptospira interrogans* serovar Canicola, supporting the potential for dogs to act both as reservoirs and incident hosts for this serovar. Across the entire study period, multiple independent infection sources seemed likely based on molecular and spatial epidemiologic analysis, which might have included exposure to other dogs in indoor congregate facilities, rodents, or rodent urine. Our findings support the need for vaccination of dogs to reduce the risk to dog and human health, a high index of suspicion for the disease in unvaccinated dogs, the combined use of molecular and serologic tests to optimize diagnosis, and early treatment to optimize outcomes.

## INTRODUCTION

Leptospirosis is a multisystemic zoonotic disease of humans, domestic dogs, and other mammalian host species caused by pathogenic spirochete bacteria of the genus *Leptospira*. The spirochetes are chronically shed in the urine of a variety of subclinically infected reservoir hosts, especially rodents (1–3). Several different serovars of *Leptospira interrogans* and *Leptospira kirschneri* have been identified in dogs with leptospirosis, including serovar Canicola (4–7), for which domestic dogs have also been considered the primary reservoir host. Clinically, severe leptospirosis in dogs resembles Weil’s disease in humans; signs include fever, lethargy, inappetence, polydipsia, polyuria, vomiting, diarrhea, and icterus due to acute kidney injury (AKI) and cholestatic hepatopathy (1).

Worldwide, leptospirosis is more prevalent in subtropical regions with heavy rainfall. In the United States, seasonality in dogs is associated with regional precipitation patterns (1, 8, 9). In both humans and dogs, most infections occur when spirochetes in contaminated water or soil penetrate intact mucous membranes or abraded skin. Less often, direct transmission can follow exposure to contaminated urine from reservoir hosts, bite wounds, venereal transmission, or predation (1, 10–12). Most cases are sporadic, but outbreaks in humans often follow flooding or sporting events involving water (13). Transmission between incidental hosts is rarely described, perhaps because of reduced shedding compared to reservoir hosts (14). For almost a century, dogs have been implicated as an important reservoir for transmission of *L. interrogans* serovar Canicola to humans, especially in resource-poor countries where free-roaming dogs are widespread (1, 15–18), although molecular proof of transmission is lacking and evidence for widespread subclinical carriage has predominantly been based on serologic data, which does not reliably predict the infecting serovar (19). Furthermore, chronic and subclinical shedding of serovar Canicola has also been identified in other species, including rodents, pigs, and cattle (16, 20–22). Before 2023, vaccination of dogs for leptospirosis was reserved for dogs considered at risk based on geographic region and lifestyle (1). Current vaccines for dogs are serovar-specific bacterins; in North America, these include serovars Canicola, Icterohaemorrhagiae, Grippotyphosa, and Pomona.

Diagnosis of leptospirosis is optimized by combining serologic tests and pathogen-detection tests (real-time PCR and culture) (23). Acute and convalescent phase serologic testing using the microscopic agglutination test (MAT) is widely accepted as the gold standard test in humans and dogs, but is laborious to perform, often negative in acutely ill patients, and requires maintenance of a large panel of live pathogenic *Leptospira* serogroups to optimize sensitivity (23). Culture requires special media, expertise, and often weeks or months of incubation, so it is not widely used for routine diagnosis; however, it is required for accurate serovar identification. Because spirochetes are found in the blood during the first week of illness, and later appear in urine, testing both blood and urine using PCR optimizes clinical sensitivity (23), although the relative clinical sensitivity of blood versus urine PCR is poorly understood. In North America, point-of-care lateral-flow chromatographic assays are commercially available for the detection of antibodies to pathogenic leptospires in dogs (e.g., IgM [WITNESS Lepto Rapid Test, Zoetis, Parsippany, NJ], IgG and IgM [SNAP Lepto, IDEXX, Portland, ME]) (1). To date, clinical validation of these assays has been limited, and assay performance might vary regionally depending on circulating serovars.

In Los Angeles (LA) County, California, laboratories and veterinarians must report leptospirosis to local public health authorities (24). In 2021, the LA County Department of Public Health (DPH) identified an outbreak of leptospirosis involving at least 201 client-owned dogs in west LA (24). Many dogs developed illness within days of being boarded in one of two regional dog daycare facilities in the region, although specific details were not available. Because affected dogs often seroconverted with very high MAT titers to serogroup Canicola, a serovar belonging to serogroup Canicola was suspected as the cause. It was therefore proposed that the outbreak was fueled by dog-to-dog transmission via direct contact with infected urine (25). Within a 6-month period, more than 50 affected dogs were examined at two major veterinary specialty hospitals in the region, where extensive diagnostic workups and treatment occurred. We sought to use this unique opportunity to accurately identify the causative agent and characterize risk factors, and describe clinical findings, diagnostic test results, and outcomes.

## MATERIALS AND METHODS

### *Leptospira* isolation and identification

At the time of the outbreak (September 2021), we contacted veterinarians at two veterinary emergency and specialty practices in West LA, located 0.6 km apart: VCA Animal Specialty and Emergency Center (ASEC) and VCA West LA Animal Hospital (WLA). We requested blood and urine specimens from dogs that were highly suspected to have leptospirosis for concurrent *Leptospira* culture, real-time PCR, and serologic testing. Dogs were excluded from this part of the study if antimicrobial drug therapy had commenced. In preparation for specimen collection, 5-mL aliquots of *Leptospira* Hornsby-Alt-Nally (HAN) (26) liquid media, HAN semi-solid media, and T80/40/LH semi-solid media (26) were shipped to WLA overnight on ice. Uninoculated media were stored at 4°C. Specimens were collected by cystocentesis or direct venipuncture. In October and November 2021, to optimize the likelihood of successful culture (27), semi-solid media were inoculated at point-of-care with 2–3 drops of either urine or blood from four dogs (LAD1 and LAD3–LAD5). Liquid HAN media was inoculated with 1 mL of urine but not blood. Inoculated media, blood, and urine were immediately shipped at ambient temperature to the National Centers for Animal Health (United States Department of Agriculture, Ames, IA). There, aliquots of media were subjected to direct fluorescent antibody (DFA) examination and *Leptospira* culture using HAN liquid (37°C), HAN semisolid (37°C), and T80/40/LH semisolid media (29°C). Blood and urine were subjected to real-time *lipL32* gene PCR (28, 29). Inoculated tubes were examined daily by darkfield microscopy. Isolates were subjected to whole-genome Illumina sequencing, and serotyping was performed using microscopic agglutination as previously described (30). Serovar typing of strains LAD1, LAD3, LAD4, and LAD5 was performed at the WOAH Reference Laboratory for Leptospirosis, University of Amsterdam, the Netherlands, by the MAT with panels of monoclonal antibodies that characteristically agglutinate serovars from the serogroup Canicola, as previously described (31).

### *Leptospira* genome enrichment

#### DNA capture and enrichment methods

For one dog (LAD2), inoculated media were not provided, but *lipL32* gene real-time PCR was positive on urine (Ct = 26.3). To obtain a genome from this sample, we performed *Leptospira* genome capture and enrichment, as previously described (32, 33). Extracted DNA was diluted to ~4 ng/µL in 10 mM Tris-HCl to a final volume of 40 µL and sonicated to an average size of 162 bp using a Q800R2 sonicator (QSonica, Newtown, CT, US). A dual-indexed library was then prepared using Agilent Sure-Select methodology (XT-HS kit, Agilent, Santa Clara, CA, US). One round of DNA capture and enrichment was performed. The enriched library was sequenced on an Illumina MiSeq instrument using a MiSeq v3 600-cycle kit (2 × 300 base pair reads).

#### Read classifications

To estimate the percentage of *Leptospira* reads in the enriched sequences, reads were mapped against the standard Kraken database with Kraken v2.1.2 (34).

#### Genome assemblies, read mapping, and phylogenomics

**Fig 1:**
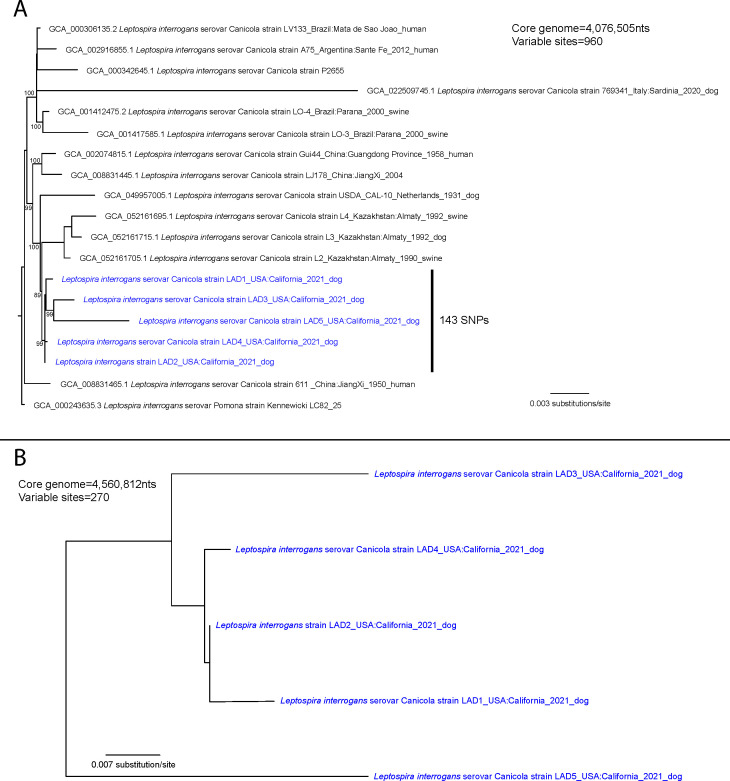
*Leptospira interrogans* serovar Canicola whole-genome phylogenies. (**A**) Whole-genome maximum likelihood phylogeny of four *Leptospira interrogans* serovar Canicola isolate genomes and one enriched genome from dogs (blue text) with 13 publicly available *Leptospira interrogans* serovar Canicola genomes based upon a concatenated SNP alignment of 960 variable positions within a shared core genome of 4,076,505 nucleotides (nts). The phylogeny was rooted with reference genome *L. interrogans* serovar Pomona strain Kennewicki LC82_25. The canine isolates group together into a unique clade defined by three novel SNPs and displaying 99% bootstrap support (bootstrap values indicated on branch nodes). (**B**) Whole-genome maximum likelihood phylogeny based upon an alignment of 270 variable nucleotide positions within a shared core genome of 4,560,812 nts using five dog genomes generated from isolates and via DNA capture and enrichment. The phylogeny was midpoint rooted and illustrates the full SNP diversity within the core genome of these infecting strains.

Sequencing reads were assembled for LAD1 and LAD3–LAD5 using SPAdes v3.13.0 (35) with default settings, and the multi-locus sequence type (ST) was determined by querying the assemblies against the pubMSLT (scheme 1) database (36). Two phylogenies were created. First, single-nucleotide polymorphisms (SNPs) were identified among the four isolate genomes (LAD1, LAD3, LAD4, and LAD5), one enriched genome (LAD2), and 13 publicly available *L. interrogans* serovar Canicola genomes (GenBank accession numbers provided in Fig. 1) by aligning LAD2 enriched reads against the LAD1 assembly using minimap2 v2.22 (37). SNPs were called from the BAM file with GATK v4.2.2 (38) using a depth of coverage ≥10× and a read proportion of 0.9. SNPs that fell within duplicated regions, based on a reference self-alignment with NUCmer v3.1 (39), were filtered from downstream analyses. All methods were wrapped by NASP v1.2.1 (40). Maximum likelihood phylogenies were then inferred on the concatenated SNP alignments using IQ-TREE v2.2.0.3 with default parameters, 1,000 bootstrap replicates (41), and the integrated ModelFinder method (42). The phylogeny was rooted with *L. interrogans* serovar Pomona strain Kennewicki LC82_25 (GCA_000243635.3). To determine the breadth of coverage, enriched reads were aligned against the reference genome *L. interrogans* serovar Pomona strain Kennewicki LC82_25 with minimap2, and the per-base depth of coverage was calculated with Samtools v1.6 (43). Second, to investigate fine-scale differences among the five dog genomes (LAD1-5), a maximum likelihood phylogeny was created as described above, except using the “-fast” option in IQ-TREE, and only LAD1-5 genomes were included (LAD1 was used as the reference). This phylogeny was midpoint rooted.

### Medical records review

In 2022, after the outbreak concluded based on reporting data gathered by the LA County DPH (24), we performed a search of electronic medical records from ASEC and WLA for client-owned dogs diagnosed with leptospirosis from April 2021 to December 2021 (inclusive). Each of the search terms “lepto*,” “microscopic agglutination test,” “MAT,” and “lepto* PCR” was used. To be included in the study, dogs had to have two or more of the following clinical signs consistent with leptospirosis: lethargy, polyuria or polydipsia, hyporexia or anorexia, vomiting, or diarrhea. In addition, diagnosis of leptospirosis required detection of *Leptospira* DNA in blood or urine using PCR, a single MAT titer ≥1:6,400, or a fourfold or greater rise in MAT titers. Real-time PCR was performed at either IDEXX Laboratories (North Grafton, MA, USA) (ASEC) or Antech Diagnostics (WLA). Serologic testing using MAT was performed at either IDEXX Laboratories (ASEC) or Michigan State University (through Antech Diagnostics) (WLA) using their standard laboratory testing protocol for diagnostic specimens covering six serovars: Autumnalis, Bratislava, Canicola, Grippotyphosa, Icterohaemorrhagiae, and Pomona. Both laboratories participate in the International Leptospirosis Proficiency Testing Scheme (44).

Information extracted from medical records included *Leptospira* vaccination history; zip code of residence, date of initial examination, and duration of illness; age (years), sex, breed, and body weight (kg); clinical signs; the results of hematology, serum biochemistry, and urinalysis (hematocrit; neutrophil count; platelet count; serum concentrations of urea nitrogen, creatinine, and total bilirubin; serum activities of ALT and ALP; presence or absence of glucosuria); results of *Leptospira* real-time PCR and serologic testing using MAT, WITNESS Lepto (Zoetis), and SNAP Lepto (IDEXX Laboratories); duration of hospitalization; antimicrobials administered for treatment of leptospirosis; and outcome (survival, death, or euthanasia). Based on the frequency of case presentations at the two hospitals, case data were organized into three periods relative to the peak of the epidemic curve (pre: 4/17/2021 to 6/4/2021, peak: 7/8/2021 to 9/8/2021, and post: 9/20/2021 to 12/5/2021).

### Statistical analysis

Univariate analysis was used to compare case variables (age, breed, sex, body weight) to those of the background hospital population (hereafter referred to as controls). Controls consisted of dogs examined at ASEC and WLA between April and December 2021 (inclusive). To protect confidentiality, data for controls were only available in categorical format. For continuous case data, normality was determined using a Shapiro-Wilk test. Categorical variables were compared using Fisher’s exact test. A Mann-Whitney U test was used to assess the relationship between serologic test results and duration of clinical signs. The Wilcoxon matched pairs signed rank test was used to compare the initial serum creatinine concentration to the serum creatinine concentration after treatment. All analyses were performed using GraphPad Prism v10.4.1; *P* values ≤ 0.05 were considered significant.

Spatial analysis was performed using ArcGIS Pro 3.6.0 (Esri Inc., Redlands, CA). The spatial distribution of cases per 100,000 households was compared to that for controls at the zip code of residence level. Household data for zip code tabulation areas for 2021 were obtained from the United States Census Bureau. Zip code areas within a 15–20 km radius of the clinics (zip code 90025) were used for analysis, which included 57/58 (98.3%) of the leptospirosis cases from California and 15,536/23,731 (65.5%) of the hospital patient population. Global Moran’s I was used to assess for spatial autocorrelation, where a positive Z-score and significant *P* values indicate spatial clustering. The Getis-Ord Gi* statistic (fixed distance band or inverse distance methods) was used to identify statistically significant spatial clusters of cold spots (low) and hot spots (high) across the entire outbreak, as well as by pre-peak, peak, and post-peak periods. Hot spots were compared for hospital patients and cases using a Hot Spot Analysis Comparison.

## RESULTS

### *Leptospira* isolation and identification

*Leptospira interrogans* serovar Canicola ST34 was isolated in media from four of the five dogs (LAD1 and LAD3–5) from which blood and urine were submitted for culture. All isolates were obtained from urine. The results of DFA, real-time PCR, and culture are shown in Table 1. Specimen collection dates, zip codes of residence, and exposure histories for the affected dogs are shown in Table S2. For the LAD2 urine specimen, after one round of enrichment, 99.4% (1,456,535/1,464,826) of sequencing reads were assigned to *Leptospira* with a breadth and depth (minimum 10×) of coverage across the reference genome (*L. interrogans* serovar Pomona strain Kennewicki LC82_25) of 96.1% and 67.8%, respectively. Phylogenetic analyses revealed that leptospires in these five dogs (four genomes from cultures and one enriched genome) shared a common ancestry (as indicated by the shared presence of three novel SNPs), but were not identical, displaying 143 SNPs among them (Fig. 1A). When comparing the core genome among the five isolates (4,560,812 nucleotides), we identified 12–224 SNPs between them (Fig. 1B). LAD1 and LAD2 were the most similar (separated by 12 SNPs and both from the West Hollywood region).

**TABLE 1:** Results of direct fluorescent antibody, PCR, and culture in three different media types for *Leptospira* spp. on five dogs suspected to have leptospirosis during an outbreak of leptospirosis in Los Angeles County^*a*^^,^^*b*^

Specimen	DFA+	PCR+ (Ct)	HAN liquid	HAN SS	T80/40/LH SS
Blood	0/2	1/5 (38.9)	0/3	0/3	0/3
Urine	2/3	5/5 (ND, 25, 25.7, 26.3, 31)	3/5 (PID 2)	4/5 (PID 2–12)	4/5 (PID 2–19)

^*a*^ DFA, direct fluorescent antibody; Ct, cycle threshold value; SS, semisolid; ND, not done; PID, post-inoculation day when spirochetes were identified by darkfield microscopy.

^*b*^ Results are expressed as number positive/number of dogs tested.

### Risk factors

**Fig 2:**
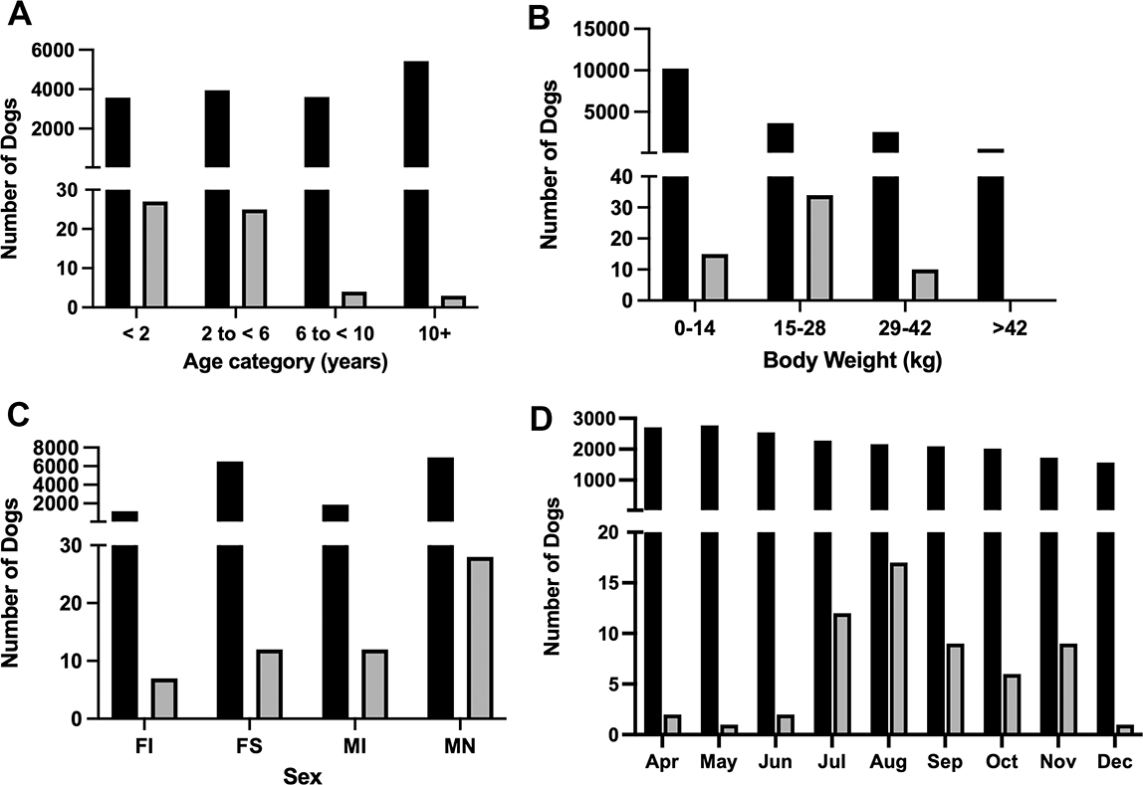
Age (**A**), body weight (**B**), sex (**C**), and month of evaluation (**D**) of 59 dogs with leptospirosis (gray bars) evaluated at two west Los Angeles specialty veterinary practices between 1 April and 31 December 2021, compared to those for the combined background hospital populations (black bars).

Fifty-nine dogs met the inclusion criteria: 23 from ASEC and 36 from WLA. When compared with the controls (15,536 dogs), cases had higher odds of being <6 years of age (OR 8.41 [CI 3.91–18.09], *P* < 0.001), ≥15 kg (OR 4.35 [CI 2.44–7.76], *P* < 0.001), and male (OR 1.78 [CI 1.04–3.06], *P* = 0.04) (Fig. 2A through C). Among female dogs, cases had higher odds of being intact compared to control dogs (OR 3.35 [CI 1.35–8.31], *P* = 0.02). There was no effect of intact status for males (OR 1.63 [CI 0.83–3.16], *P* = 0.18). Golden Retrievers (5/59, *P* < 0.02) and Siberian Huskies (3/59, *P* < 0.01) were overrepresented in the case population. Possible sources of exposure were identified in 45/59 (76%) dogs (Table S3). Thirty-one of 59 (53%) had exposure to indoor congregate facilities (ICFs); 24 (77%) of these 31 dogs were examined during the peak period. Time between last known exposure to ICFs and onset of signs for 19 of the 30 dogs ranged from 2 to 35 days (median, 5 days). Cases in the peak period had higher odds of boarding or daycare than cases outside this window (OR 5.7 [CI 1.78–19.25], *P* < 0.01). Among cases, we found no association between sex and breed (Siberian Husky or Golden Retriever) and exposure to ICFs, but dogs <6 years of age (OR 10.00 [CI 1.09–120.9], *P* = 0.03) and dogs ≥15 kg were more likely to have exposure to ICFs than dogs <15 kg (OR 5.52 [CI 1.46–18.24], *P* = 0.02).

Of 47 dogs with a known *Leptospira* vaccination history, vaccines had never been administered to 41/47 (87.2%) dogs; 6/47 (12.8%) dogs had received a leptospirosis vaccine (all with Nobivac Lepto4, Merck Animal Health, NJ). Of the vaccinated dogs, four had only received the first of a 2-dose initial vaccination series before onset of illness and were not yet due for the second dose; one received the second dose 5 days before illness onset, and another 4 days after illness onset. The diagnosis for dogs that had received a vaccine was made using real-time PCR on urine (5) or blood (1); one of the dogs with a positive urine PCR had a positive MAT (1:25,600) 9 days after its initial vaccine dose.

**Fig 3:**
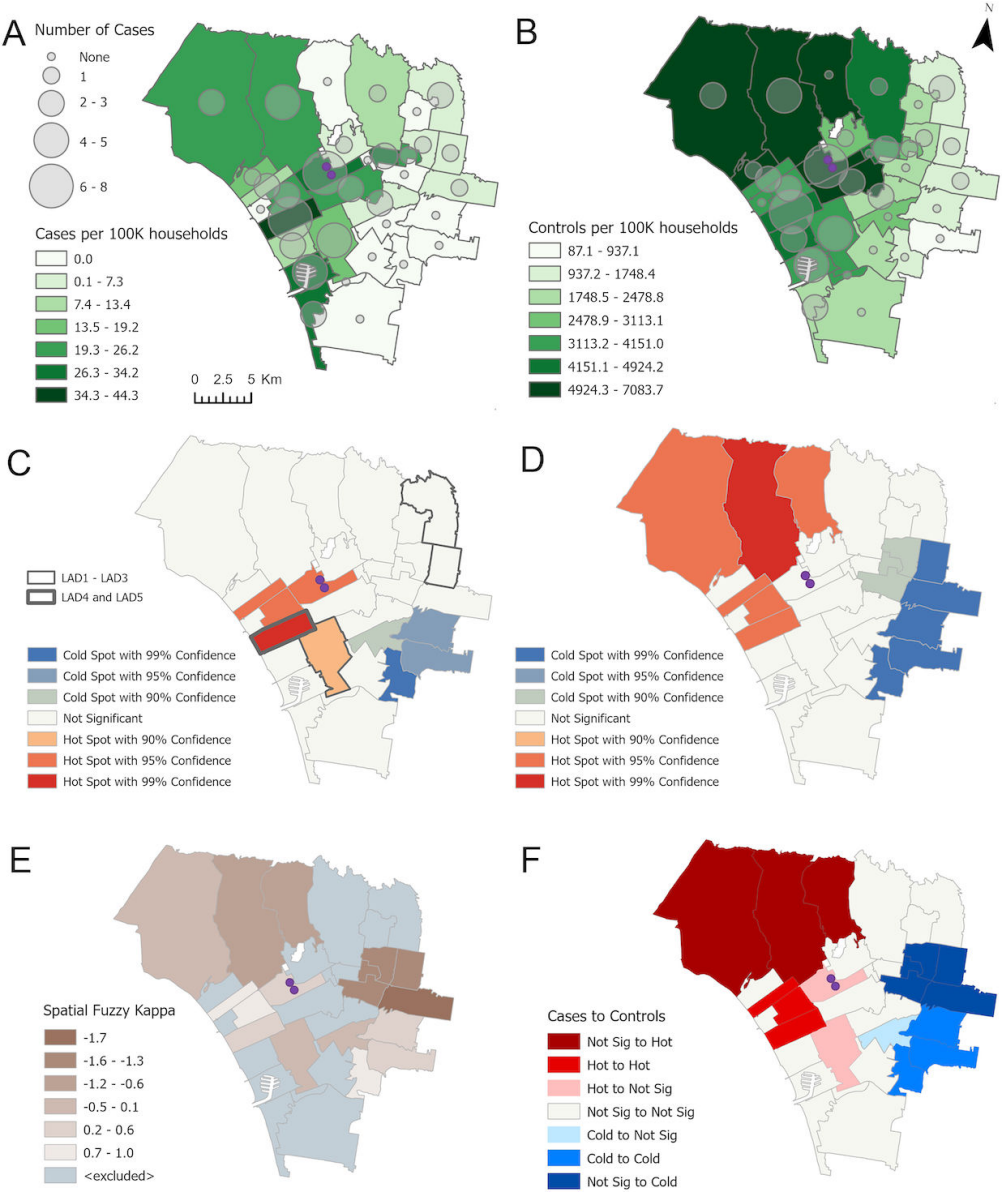
Maps of the Los Angeles area used for spatial analysis (zip code regions within a 15 km radius of two veterinary specialty clinics [purple circles]). Graduated fill colors in zip code regions represent incidence data (cases and background hospital population [controls] per 100,000 households); transparent circles represent absolute case numbers. (**A**) Leptospirosis cases in the study area (*n* = 57; two dogs fell outside the study area). (**B**) Controls (*n* = 15,536). (**C and D**) Distribution of hot spots and cold spots for case incidence and control incidence, respectively. In panel **C**, regions from which isolates were obtained are shown; the most similar isolates (LAD1 and LAD2) were from contiguous eastern zip codes. LAD4 and LAD5 were both from the same region in Santa Monica. (**E and F**) Spatial fuzzy kappa (**E**) and significance comparisons (**F**) of hot and cold spots for case to control incidence. Hot spots (or cold spots) that are perfectly associated between cases and control populations have spatial fuzzy kappa values of 1. Negative values indicate a negative relationship; negative values have no lower bound, but values are rarely as low as −3. The significance comparison compares significance categories between cases and the control population. Cold-to-hot, cold-to-nonsignificant, and hot-to-cold regions were not identified. Hot spots for cases are significantly distributed south and west of the clinic zip code region, whereas those for the hospital background population also include northern and eastern zip code regions. Sources: US Census Bureau; VCA, Inc.

Thirty-four (58%) of cases were evaluated during the peak period. The temporal (Fig. 2D) and spatial (Fig. 3) distribution of cases differed from those of the control population, with cases clustering in zip code regions west and south of the clinics. We detected significant autocorrelation for cases (*P* = 0.01, Z-score = 2.5) and for controls (*P* < 0.001, Z-score = 5.6). Case hotspots shifted from regions concentrated around the clinics to regions southwest of the clinics during and after the peak of the epidemic, respectively (Fig. S1).

### Clinicopathologic findings

Clinical signs documented for >10% of affected dogs were lethargy (45/59 [76%]), hyporexia (32/59 [54%]), vomiting (28/59 [47%]), polyuria/polydipsia (19/59 [32%]), anorexia (12/59 [20%]), and diarrhea (10/59 [17%]). Median duration of signs at initial examination was 3 days (range, 0–30 days). Laboratory abnormalities included anemia, neutrophilia, thrombocytopenia, azotemia, and biochemical evidence of cholestatic hepatic injury (Table 2). When results of both renal and hepatic function were available, 6/40 (15%) dogs had evidence of concurrent renal and liver function impairment, and 29/40 (73%) had only evidence of renal function impairment; none had evidence of liver function impairment alone (Table S4). Urinalysis revealed glucosuria in 15/39 (38%) dogs. Seven dogs had thoracic radiographs; initial radiographs revealed no abnormal findings (*n* = 4) or mild diffuse interstitial to bronchointerstitial patterns (*n* = 3). For two dogs, follow-up radiographs were available; both had normal initial radiographs. One had a mild, diffuse interstitial pattern 2 days later; the other had a marked diffuse interstitial to alveolar pattern 7 days later that was consistent with leptospiral pulmonary hemorrhage syndrome.

**TABLE 2:** Hematologic and biochemical findings in 59 dogs diagnosed with leptospirosis during an outbreak in Los Angeles County^*a*^

	Hct (%)	Neutrophils (/uL)	Platelets (/uL)	BUN (mg/dL)	Creat (mg/dL)	ALP (IU/L)	ALT (IU/L)	TBili (mg/dL)
Reference interval	39–58	2,060–10,600	170,000–400,000	7–26	0.4–1.3	5–131	12–118	<0.1–0.3
*n*/Number tested (%)	29/56 (52%)	20/41 (49%)	13/38 (34%)	47/59 (80%)	52/59 (88%)	7/40 (18%)	5/40 (13%)	6/40 (15%)
Range	23–55	7,128–30,576	43,000–597,000	12–200	0.7–33.3	13–1,137	13–1,427	<0.1–11.8
Median	38	14,560	199,500	41	3.5	57.5	35.0	0.2
Mean	38.6	15,257	233,054	55.5	4.6	117.1	95.8	0.7

^*a*^ *n*, Number of dogs with results outside the reference interval; Hct, hematocrit; BUN, blood urea nitrogen; Creat, creatinine; ALP, alkaline phosphatase; ALT, alanine transaminase; TBili, total bilirubin.

### Nucleic acid amplification testing

**Fig 4:**
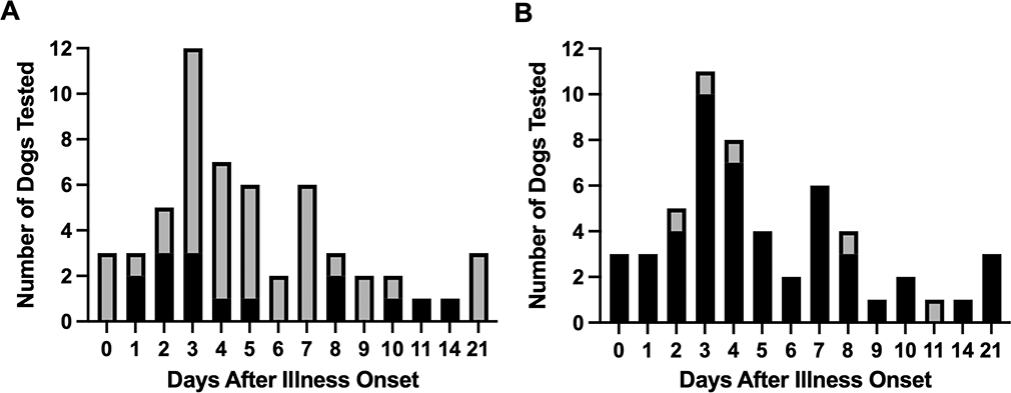
Results of *Leptospira LipL32* real-time PCR testing on blood (**A**) and urine (**B**) in 56 and 54 tested dogs with leptospirosis, respectively, plotted against the day of testing relative to illness onset. Black bars = number of dogs with positive results; gray bars = number with negative results.

*Leptospira lipL32* gene real-time PCR was positive on whole blood in 15/56 (27%) dogs and urine in 49/54 (91%) dogs (OR 0.04, 95% CI 0.01–0.11, *P* < 0.001) (Table 3; Fig. 4). In 52 dogs that had both blood and urine tested, nine (17%) had positive PCR results for both urine and blood.

**TABLE 3:** Median (range) days from illness onset to diagnostic test results in 59 dogs diagnosed with leptospirosis during an outbreak in Los Angeles County^*c*^

Assay	*n*	Median (days)	Range (days)
Initial MAT			
All	29	5	0–30
Highest ≥1:6,400^*a*^	13	5	0–30
Negative (<1:50)	7	3	1–7
Highest 1:100 to 1:3,200	9	9	4–22
Convalescent MAT ≥4-fold increase or single ≥1:6,400	19	21	4–49
WITNESS Lepto Rapid			
All	27	4	1–10
Positive^*b*^	20	4	1–9
Negative	7	3	2–10
SNAP Lepto			
All	9	6	1–21
Positive^*a*^	8	6.5	3–21
Negative	1	1	–
PCR			
All	58	4	0–21
All blood	56	4	0–21
Positive blood	15	3	1–14
Negative blood	41	4	0–21
All urine	54	4	0–21
Positive urine	49	4	0–21
Negative urine	5	4	2–21

^*a*^ One dog received the first dose of a leptospirosis vaccine 9 days before testing, but was also PCR-positive on urine.

^*b*^ One dog received the first dose of a leptospirosis vaccine 10 days before testing, but was also PCR-positive on urine.

^*c*^ MAT, microscopic agglutination test.

### Serologic testing using MAT

Serology using MAT was performed in 29/59 (49%) dogs, only one of which had recently received a leptospirosis vaccine (Table 3; Table S5). All seven dogs with negative MAT test results were tested from 1 to 7 days after the onset of illness. Of 20 dogs tested in the first week of illness, nine had highest titers ≥1:6,400; seven had negative titers. Nineteen (66%) of the 29 dogs tested had either a single highest titer ≥1:6,400 (*n* = 8) or acute and convalescent testing (*n* = 11) with the highest titers to serovar Canicola; lower titers were variably identified to other serovars tested. The acute to convalescent testing interval was 7 to 36 days (mean, 21 days; median, 23 days). Seroconversion was documented in six dogs. The remaining dogs had both high (>1:6,400) acute and convalescent titers, but acute and convalescent titers were either reported as the same or one dilution lower.

### Serologic testing using point-of-care assays

Serum antibodies to *Leptospira* were detected in 20/27 (74%) dogs using the WITNESS Lepto Rapid Test (one dog had received a leptospirosis vaccine 10 days previously) and 8/9 (89%) dogs using the SNAP Lepto; one dog had both tests performed and was positive on both assays. Median (range) days of illness to test results are shown in Table 3. With the exception of one dog, all dogs with negative test results had been sick for ≤7 days; the exception was a dog with a negative WITNESS test after 10 days of illness. This dog had a positive PCR result on blood, and illness began as intermittent vomiting and inappetence, then transitioned to increased thirst and urination. Other serologic tests were not performed in this dog.

**Fig 5:**
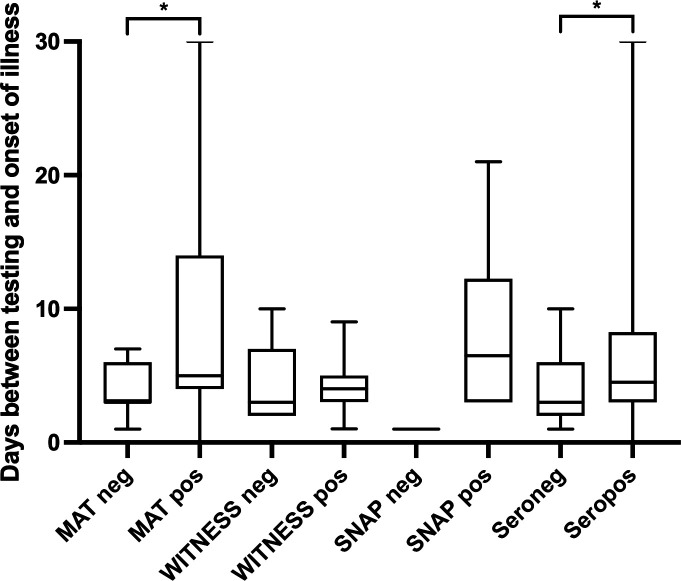
Box-and-whisker plots showing days from onset of illness to testing for MAT, the WITNESS Lepto Rapid test, the SNAP Lepto test, and all serologic test results in dogs with leptospirosis. neg = negative, pos = positive, * significant difference (0.01 < *P* < 0.05). Whiskers represent the minimum to maximum values.

Using the case definition as the gold standard, the sensitivity of blood and urine PCR for *Leptospira* DNA was 27% and 91%, respectively. The sensitivity of point-of-care assays (WITNESS Lepto Rapid Test and SNAP Lepto) for the detection of antibodies to pathogenic leptospires was 74% and 89%, respectively. For initial MAT and when all serologic test types were combined, dogs with negative test results had a significantly shorter duration of illness than those with positive test results (median, 3 days versus 4 days, *P* = 0.02) (Fig. 5). Table S6 shows the results of PCR versus serology for dogs that had both blood and urine PCR and any serologic test performed.

### Treatment and outcome

Seventy-three percent (43/59) of dogs were hospitalized; 16 (27%) were treated as outpatients with oral doxycycline. Median duration of hospitalization was 4 (range, 1–15) days. Hospitalized dogs were treated with intravenous (IV) fluids and antibacterial drugs (IV ampicillin or ampicillin-sulbactam [*n* = 14], IV doxycycline [*n* = 14], oral doxycycline [*n* = 12], or a combination of ampicillin/sulbactam and doxycycline [*n* = 3]). Two dogs were treated with hemodialysis (nine treatments each). All dogs that lived to discharge (54/59 [92%]) were then treated with oral doxycycline for 7–30 (median 14, mean 15) days. For dogs that survived to follow-up, there was a progressive and significant reduction in median serum creatinine concentration from initial evaluation to discharge, then discharge to follow-up (both *P* < 0.01) (Table 4; Fig. S2A and B). The two dogs that were dialyzed had the highest initial serum creatinine concentrations (19.0 and 33.3 mg/dL); at last follow-up, these were 1.9 and 2.9 mg/dL, respectively. International Renal Interest Society (IRIS) AKI grades (45) based on the last serum creatinine concentration available for dogs that lived and dogs that were euthanized are shown in Fig. S2C (*P* < 0.001). The dog with IRIS Grade I AKI that was euthanized had suspected severe leptospiral pulmonary hemorrhage syndrome. The remaining four dogs that were euthanized had moderate to severe azotemia (IRIS AKI grades IV or V).

**TABLE 4:** Median (range) of serum creatinine concentrations at initial evaluation, discharge, and follow-up relative to onset of illness in dogs diagnosed with leptospirosis during an outbreak in Los Angeles County

Population (number of dogs)	Timing of serum creatinine measurement	Serum creatinine concentration (mg/dL)	Timing relative to onset of illness (days)
All dogs (58)	Initial	3.5 (0.7–33.3)	4 (0–30)
Dogs with creatinine measured at discharge (40)	Initial	3.8 (0.7–19.0)	4 (1–21)
	Discharge	2.4 (0.9–14.8)	9 (3–27)
Dogs with creatinine measured at a follow-up visit (27)	Initial	4.1 (1.6–33.3)	4 (0–21)
	Discharge (*n* = 23)	2.4 (1.3–12.5)	9 (5–27)
	Follow-up	1.6 (0.9–5.2)	42 (8–210)

## DISCUSSION

The results of this study offer significant advancements in our understanding of the epidemiology, diagnostic test performance, and treatment outcomes of leptospirosis in dogs, and shed light on the value of vaccination to reduce the potential public health risk of dog ownership. The existence of an outbreak was supported by the temporal and spatial clustering of cases. During the peak period, we found an association with ICF exposure. Although whole-genome sequencing (WGS) analysis did not support dog-to-dog transmission, we could only obtain isolates during the post-peak period and only one of those isolates was from a dog with exposure to an ICF (boarding). However, many dogs had no history of ICF exposure or close contact with other dogs. Although accurate data on the location of ICFs, duration of exposure, and housing conditions in ICFs were not available, most were dog daycares (implying day-long exposures) where dogs were group-housed in close proximity with one another.

To our knowledge, this is the first study to report isolation and characterization of pathogenic leptospires from dogs from the United States since the early 1990s (15, 46). Previous studies have relied on sequencing of PCR amplicons, which lack discriminatory power and the ability to accurately identify the infecting serovar (19). All five dogs from which sequence information was available were infected with *L. interrogans* serovar Canicola ST34, but the infecting strains were distinct, suggesting multiple infection sources. Indeed, the LAD1-5 genomes represented 14.9% (143/960) of the total SNPs identified among the *L. interrogans* serovar Canicola genomes included in our phylogenetic analysis, which spanned 90 years and four continents. The two most similar genomes were from contiguous zip code regions 10 km east of the hotspot in Santa Monica; two other genomes collected 2 weeks apart from Santa Monica were less related. Infection of other dogs with serovar Canicola also seemed likely based on the highest titer to Canicola with a magnitude of ≥1:6,400 in 17/22 dogs that tested positive with MAT. Detection of this serovar in sick dogs aligns with previous observations that dogs can act as either incidental or reservoir hosts for serovar Canicola (4, 47) and supports the need for continued inclusion of serovar Canicola in vaccines for dogs in the United States. Whether serovar Canicola causes disease or persistent subclinical shedding in dogs might depend on pathogen virulence determinants and/or host factors. Through surveillance during the outbreak, the LA County DPH reported infection in six apparently healthy dogs (25). Of interest, *L. interrogans* serovar Canicola ST34 was recently identified in dogs from Mexico, including seven subclinically infected dogs and two dogs with fatal leptospirosis (4). No known travel history to Mexico was reported for any of the dogs in this study.

We identified temporal clustering of cases in July and August 2021. Although leptospirosis often occurs 1 to 3 months after heavy rainfall or flooding (48), rainfall in LA County from January to July 2021 was not above average (49). Another possible explanation for the timing of the outbreak is overcrowding of dog daycare facilities secondary to the rise in pet ownership during the COVID-19 pandemic, followed by return-to-work activity that was documented in Los Angeles in mid-2021 (50). Concurrently, there was an outbreak of H3N2 influenza virus infection that involved over 1,300 dogs in West LA (51); such outbreaks typically follow importation of infected dogs from South Korea and China (52). A second possible pandemic-related explanation is increased rodent exposure, which occurred in large cities in the U.S. and other countries during the pandemic (53, 54). In addition, the outbreak followed a massive (17 million-gallon) offshore sewage spill into Santa Monica Bay, which occurred on 11 July 2021 (55). Although it is unlikely that this spill explained most cases based on timing and exposure histories, some dogs evaluated in the post-peak period had beach exposure in Santa Monica, where there might have been increased rodent activity due to dead wildlife or debris, although no confirmed reports of such findings were available. Other urban wildlife species seemed unlikely to be a source based on a large study of specimens collected from striped skunks, raccoons, coyotes, fox squirrels, and Virginia opossums between 2015 and 2020 (32). Using serology and WGS analysis, serovar Pomona appeared to be the predominant serovar in mesocarnivores; none of the species evaluated had significant seroreactivity to Canicola.

Historically, the risk of leptospirosis in urban dogs from Southern California has been considered low, so veterinarians were not routinely recommending leptospirosis vaccination. Evidence supports the efficacy and safety of four-serovar vaccines (1). Before four-serovar vaccines were available, dogs at risk for leptospirosis were male, young adult, medium-to-large breed outdoor dogs in rural environments or that had recreational exposure to standing water (1, 56, 57). Since the availability of four-serovar vaccines, disease has predominantly been recognized in unvaccinated small breed dogs from urban regions (1). However, dogs in the outbreak reported here had risk factors that instead resembled those of the dog population with leptospirosis before the availability of four-serovar vaccines. It is possible that male dogs are more likely to exhibit behavior that increases their risk of contact with infected urine from rodents or other dogs. Among females, we found an association with intact status; estrogen can promote some other bacterial infections (58), but we were unable to find other studies of the effect of female gonadectomy on leptospirosis. The associations with young age and higher body weight were confounded by an interaction with boarding; this might have also impacted breed associations, as 6/7 dogs that were Siberian Huskies or Golden Retrievers had exposure to ICFs. Breed associations might also reflect behavioral traits and/or breed-related immunodeficiency. Ultimately, the outbreak ended following educational efforts from the Los Angeles County (LAC) DPH regarding the need for vaccination, as well as attention to hygiene, rodent control, and pet population density in boarding facilities.

The results of diagnostic testing in this study supported the value of combining serologic test results and PCR to optimize diagnosis (23). Because serostatus does not predict infection status, and PCR must be performed before administration of antimicrobials, initial serology and PCR testing are best performed simultaneously. Consistent with the results of previous studies (59–61), with the exception of the dog with a negative WITNESS test result at day 10 of illness, all dogs with negative serologic test results had been sick for ≤7 days. For dogs that failed to seroconvert, a rise in titer might have been missed because of a long median interval between titers (23 days rather than the recommended 7–14 days) (1), or dogs might have been sick for longer than recognized by the owner. The latter seems likely based on the high titers in some dogs early in the course of illness. For some dogs that lacked serologic proof of infection, diagnosis was based on a positive PCR result; however, given that healthy dogs can be leptospiruric, positive PCR results on urine alone require cautious interpretation (1). Because the WITNESS Lepto detects IgM, positive test results using this assay in dogs with consistent clinical signs are more likely to support a diagnosis of leptospirosis than assays that detect IgG (which are more likely to be positive from previous subclinical infection) (1).

The lower sensitivity of PCR testing on blood than on urine likely reflected the transient and early nature of leptospiremia (1). However, the frequent detection of leptospires in the urine of affected dogs was unexpected, given that shedding by incidental hosts is often low-level and intermittent. The sensitivity of urine PCR might vary depending on host–pathogen interactions that influence the ability of the spirochete to persist in the renal tubules. The high prevalence of leptospiruria in this outbreak might be explained by the known adaptation of serovar Canicola to dogs, and raises concern for dog-to-dog or dog-to-human transmission. However, no evidence of human disease was documented by the LA County DPH (25).

Although most dogs survived to discharge, renal function at discharge was variable, and follow-up was limited. There was progressive recovery of renal function after discharge in some dogs. Dogs that were euthanized had severe azotemia or suspected leptospiral pulmonary hemorrhage syndrome. Previous studies have reported higher mortality rates (62–64), although case numbers were low and dogs in some of these studies were not evaluated at specialty care facilities, which might have impacted outcomes. The high index of suspicion for the disease during the outbreak (both from owners and veterinarians) might have positively impacted outcomes and diagnostic test selection. The owner’s financial constraints likely also impacted outcomes and test selection. Given that some recovered dogs remain infected with pathogenic leptospires despite antimicrobial treatment (65, 66), and considering the particular ability of serovar Canicola to establish persistent infections in dogs, clinical recovery does not imply elimination of public health risk.

In summary, we were able to characterize an outbreak of leptospirosis in dogs, supporting the potential for dogs to act both as reservoirs and incident hosts for serovar Canicola. Multiple independent infection sources seemed likely based on molecular and spatial epidemiologic analysis, which might have included exposure to other dogs in ICFs, rodents, or rodent urine. Further understanding would require studies, such as characterization of isolates from rodent populations in the region. Early diagnosis using a combination of serology and PCR resulted in survival to discharge in over 90% of affected dogs. However, as this was a retrospective study that included only two referral clinics, other dogs involved in the outbreak might have died without a diagnosis, and dogs with negative initial PCR and serology results would not have been included in our study. Thus, conclusions cannot be made about the overall sensitivity and specificity of the diagnostic tests applied. Because analytical and clinical diagnostic test performance might be serovar-specific, findings might not apply to other regions or outbreak situations. Our findings support the need for widespread annual vaccination of all dogs to reduce the risk to dog and human health, a high index of suspicion for the disease in unvaccinated dogs, combined use of molecular and serologic tests to optimize diagnosis, and early treatment to optimize outcome.

## Data Availability

Sequencing data and genome assemblies are available at NCBI under BioProject ID PRJNA1377681; accession numbers are listed in Table S1.

## SUPPLEMENTAL MATERIAL

Figure S1 jcm.00240-26-s0001.docx Spatio-temporal analysis of an outbreak of leptospirosis in dogs.

Figure S2 jcm.00240-26-s0002.docx Results of serial monitoring of renal function in dogs diagnosed with leptospirosis.

Supplemental tables jcm.00240-26-s0003.docx Tables S1 to S4, and S6.

Table S5 jcm.00240-26-s0004.xlsx Diagnostic test results for dogs with leptospirosis.

ASM does not own the copyrights to Supplemental Material that may be linked to, or accessed through, an article. The authors have granted ASM a non-exclusive, world-wide license to publish the Supplemental Material files. Please contact the corresponding author directly for reuse.
